# El desafío de comunicar y controlar la epidemia por coronavirus

**DOI:** 10.7705/biomedica.5455

**Published:** 2020-03-30

**Authors:** José Moreno-Montoya

**Affiliations:** 1 Fundación Santa Fe de Bogotá Editor asociado, Biomédica Fundación Santa Fe de Bogotá

A finales de enero de 2020, se detectó en territorio chino un nuevo coronavirus (COVID-19), responsable de un brote de neumonía en la localidad de Wuhan, después de haberse observado casos sintomáticos desde finales del año anterior ^1^. Desde entonces, los rasgos característicos del brote han sido analizados minuciosamente por la comunidad internacional con base en los informes de casos nuevos a medida que avanza la epidemia, incluida la duración del período de incubación. Su distribución geográfica hasta febrero de 2020 indicaba que, por lo menos, en 23 países había casos confirmados procedentes - en la mayoría de las ocasiones- directamente del país oriental. Entretanto, la provincia de Hubei cerró sus vuelos y sus rutas de transporte público masivo ^2^ y, dado que el virus se transmite de persona a persona, dicha medida ha logrado reducir el número reproductivo (R_0_), que equivale a la velocidad de transmisión de la enfermedad, el cual se estimó inicialmente en 2,2 ^3^. No obstante, persiste la incertidumbre de la real magnitud de dicha velocidad, y se presume que hay una diferencia significativa con respecto al valor crítico de uno, ya que los casos que actualmente se detectan y se confirman deben corresponder a personas que contrajeron la infección a mediados o a finales de enero, por lo que se requiere seguir investigando.

En este contexto, más allá de las iniciativas clínicas para el tratamiento de la infección, la respuesta sanitaria pública a nivel internacional se ha centrado en el fortalecimiento de las intervenciones no farmacéuticas, incluido el seguimiento intensivo de contactos, la cuarentena de individuos potencialmente expuestos a la infección y el aislamiento de aquellos infectados o sospechosamente sintomáticos. En el proceso, la amplísima difusión mediática ha tenido un papel fundamental, por cuanto la diseminación global del riesgo se ha destacado en los medios masivos de comunicación trascendiendo la información de publicaciones especializadas. De todas maneras, las consecuencias no han sido menos aterradoras.

La aparición de una enfermedad, especialmente cuando es causada por agentes infecciosos de rápido contagio, supone una alerta seria para la sociedad y un desafío que trasciende las fronteras en un esfuerzo que requiere comprender y racionalizar el alcance y el potencial de tal amenaza. En este sentido, el carácter inmediato de algunas comunicaciones ha favorecido la construcción de un imaginario colectivo catastrófico, lo que ha permeado incluso algunos ámbitos sanitarios. Probablemente, esas especulaciones tengan razón en la medida en que, hasta el momento, no existen medicamentos antivirales ni vacunas específicas para su prevención o control ^4^. Sin embargo, así como existen dudas sobre las medidas terapéuticas farmacológicas requeridas, también las hay con respecto a la idoneidad y la efectividad de las medidas de control. Después del brote inicial por COVID-19, la prensa internacional registró el efecto que provocó el cierre de los sistemas de transporte masivo en China. Con certeza, por lo menos 40 millones de personas detuvieron sus planes de desplazamiento ^5^. El costo económico y social de dichas medidas para el control de la infección, escapa a las estimaciones epidemiológicas que se han hecho.

Esto no quiere decir que los esfuerzos sean en vano o que su impacto potencial, inconmensurable por el momento, reste validez a las intervenciones sanitarias colectivas. Al contrario, la situación exige que las agencias de salud pública, los proveedores de atención médica y el público en general, conozcan los efectos potenciales de la epidemia y se fortalezcan las acciones coordinadas, oportunas y efectivas para prevenir casos adicionales o peores resultados sanitarios. Algunos de los componentes básicos de tales estrategias son el aprovisionamiento de equipos y el establecimiento de planes especiales de comunicación e información, que permitan el seguimiento o, por lo menos, la formalización de los mensajes institucionales sobre las medidas acertadas de prevención, del impacto derivado de la infección en nuestro contexto y de las iniciativas gubernamentales relacionadas.

Diversas comunidades alrededor del mundo han impulsado, por ejemplo, la creación de páginas web específicas para el tratamiento y la actualización de la información sobre la epidemia; además, han aprovechado las redes sociales para difundir información veraz sobre el avance de la infección y las medidas básicas de cuidado, como el uso de dispositivos de barrera, el control de los viajeros y los planes locales para atender los posibles casos. Simultáneamente, los prestadores de servicios de salud adelantan campañas directas de información, ya que sus usuarios son pacientes potenciales y, además, dichas campañas son una oportunidad para derrumbar los mitos y las creencias que rodean con increíble frecuencia este tipo de situaciones epidemiológicas.

El escenario es claro. El hecho de que el público asocie la aparición de nuevos casos de coronavirus con una amenaza real y de mayor magnitud, y que las personas quieran respuestas, debe servir como un recordatorio para todas las agencias de salud pública. Cuantas más respuestas y mayor claridad puedan proporcionar los expertos, mayor será la confianza y la observancia de las recomendaciones. Es prioritario que las entidades reguladoras, los prestadores de servicios sanitarios e, incluso, las instituciones académicas del área de la salud, den prioridad a las comunicaciones como herramienta de salud pública, aboguen por la solución de los vacíos de información y garanticen la continuidad en el seguimiento del trabajo epidemiológico, de laboratorio y de prevención del virus.

Cuanto más rápida y efectiva sea la comunicación continua sobre esta o cualquier otra situación en una fase aguda, tanto más podrán ser escuchados de manera efectiva por aquellos que, al fin de cuentas, pueden contraer la enfermedad. En esta era de la información, la comunicación masiva debe constituir una herramienta formal de la salud pública para confrontar la amenaza del COVID-19, y una oportunidad de educación, aprendizaje y prevención ante futuros eventos de emergencia sanitaria.
